# Supplementary material for: Parallel and nonparallel genomic responses contribute to herbicide resistance in Ipomoea purpurea, a common agricultural weed
Source: PLoS Genet. 2020 Feb 3;16(2):e1008593. doi: 10.1371/journal.pgen.1008593 (PMC7018220; doi:10.1371/journal.pgen.1008593)

## **S1 Fig.** **No sequence differences in *EPSPS*.** Comparison of amino acid sequences of *Ipomoea purpurea* EPSPS protein sequence (bottom two sequences) with other reported EPSPS proteins in the NCBI database (gi|170783792, gi|76782198, gi|15225450, gi|257228989, gi|16751569, gi|460388790, gi|225454012, gi|374923051, gi|46095337, gi|189170087) shows no sequence variation within the region known to affect herbicide resistance (inside red outline). Non-synonymous changes (red and blue arrows) outside of this region likewise do not correlate with resistance (Table S1).


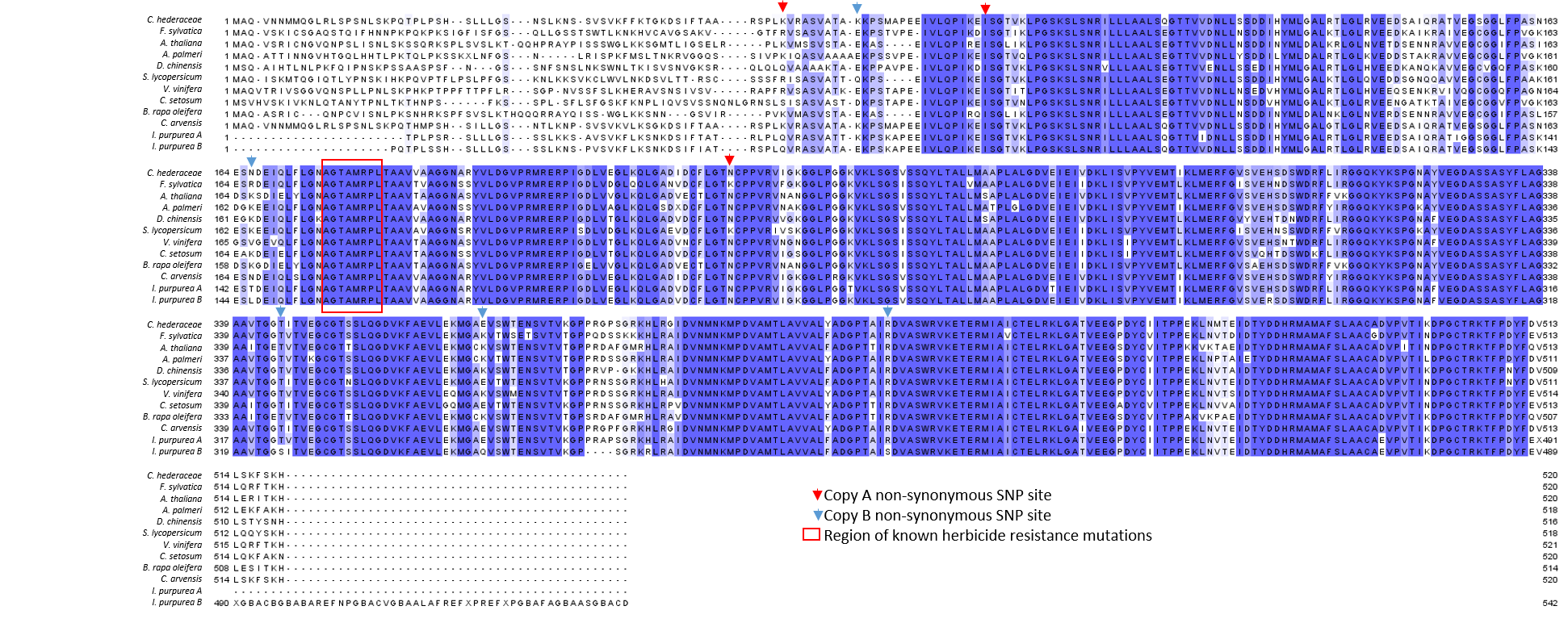

Supplement: S1 Fig — Comparison of amino acid sequences of Ipomoea purpurea EPSPS protein sequence (bottom two sequences) with other reported EPSPS proteins in the NCBI database (gi|170783792, gi|76782198, gi|15225450, gi|257228989, gi|16751569, gi|460388790, gi|225454012, gi|374923051, gi|46095337, gi|189170087) shows no sequence variation within the region known to affect herbicide resistance (inside red outline). Non-synonymous changes (red and blue arrows) outside of this region likewise do not correlate with resistance (S1 Table). (DOCX) [file pgen.1008593.s001.docx]
