# Supplementary material for: Parallel and nonparallel genomic responses contribute to herbicide resistance in Ipomoea purpurea, a common agricultural weed
Source: PLoS Genet. 2020 Feb 3;16(2):e1008593. doi: 10.1371/journal.pgen.1008593 (PMC7018220; doi:10.1371/journal.pgen.1008593)
Supplement: S2 Fig — On the tree, populations are denoted by color and tip labels; values at nodes are percent bootstrap support; population level resistance is denoted by the color in the column (red = resistant, blue = susceptible). (DOCX) [file pgen.1008593.s002.docx]

**S2 Fig. Neighbor joining tree using all of the RAD-seq SNP loci**. On the tree, populations are denoted by color and tip labels; values at nodes are percent bootstrap support; population level resistance is denoted by the color in the column (red=resistant, blue=susceptible).

**
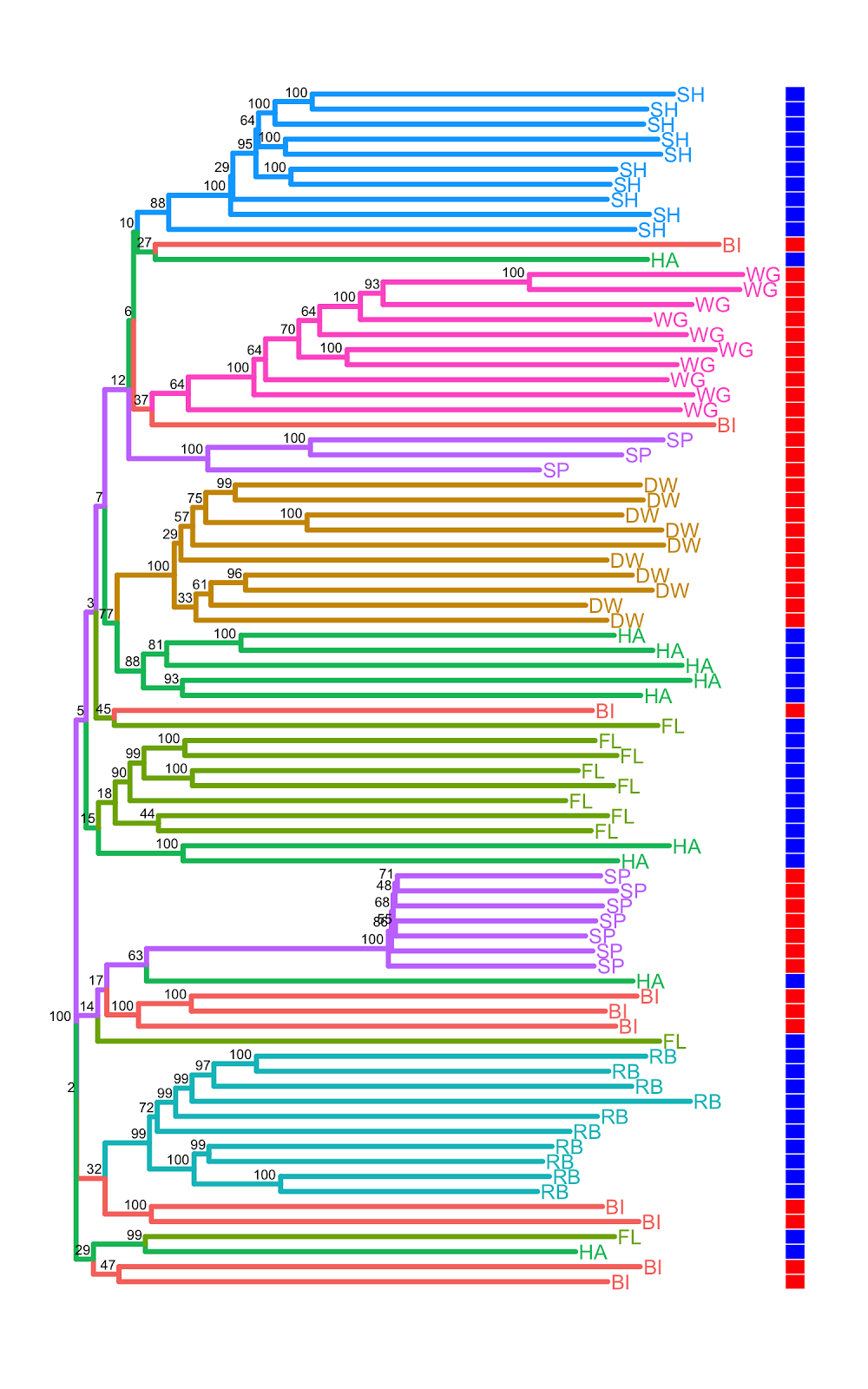
**
