# Supplementary material for: Parallel and nonparallel genomic responses contribute to herbicide resistance in Ipomoea purpurea, a common agricultural weed
Source: PLoS Genet. 2020 Feb 3;16(2):e1008593. doi: 10.1371/journal.pgen.1008593 (PMC7018220; doi:10.1371/journal.pgen.1008593)

**S3 Fig. Population structure analyses.** At K=2 FastStructure results for the RADseq data do not show the resistant populations (first four populations on the left) segregating into a distinct group, suggesting they are not from a single origin. FastStructure analysis suggests either K=6 or K=7 as the best model, both of which leads to some populations being highly admixed (*e.g.* BI) while others are fairly homogenous (*e.g.* SH).


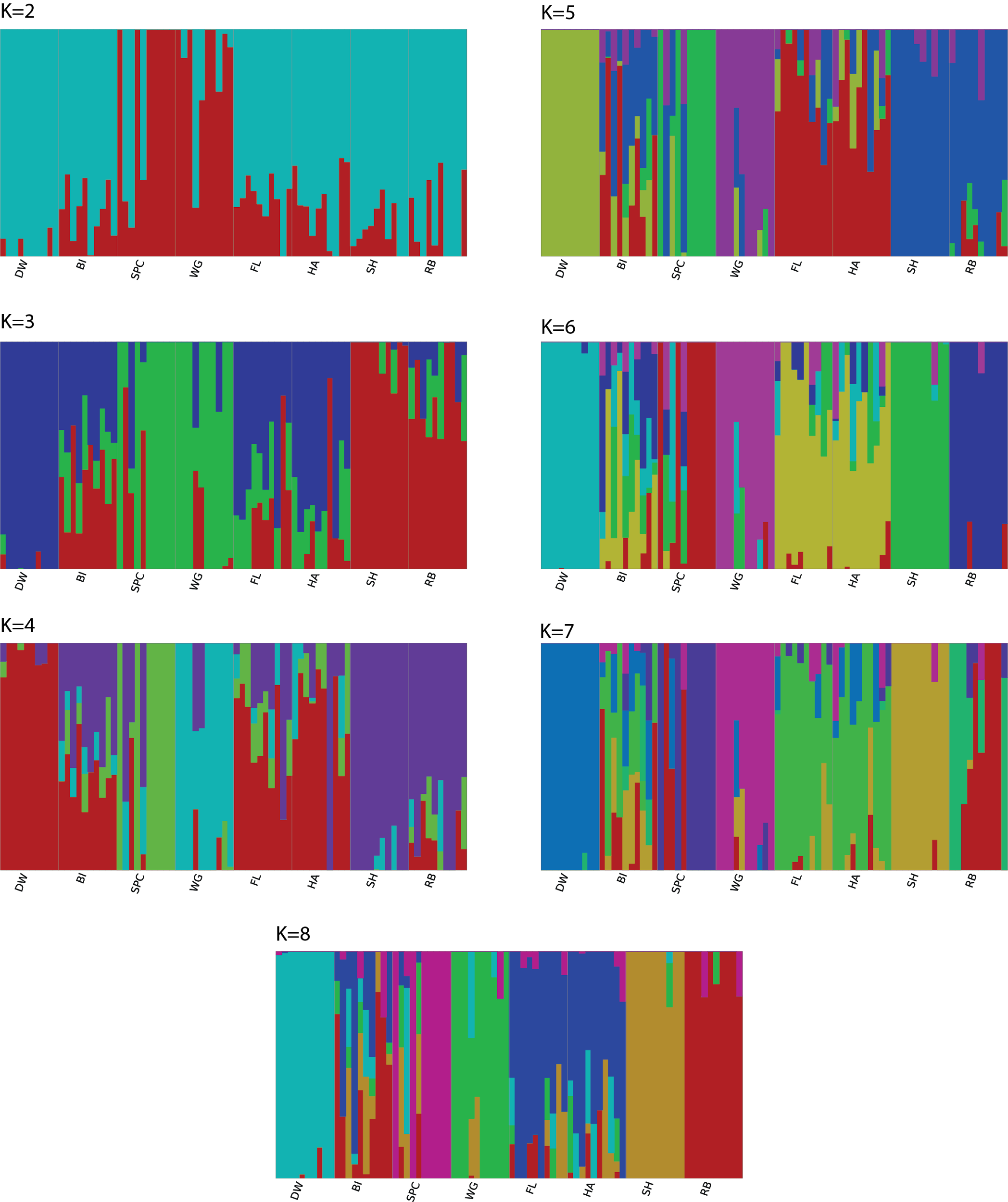

Supplement: S3 Fig — At K = 2 FastStructure results for the RADseq data do not show the resistant populations (first four populations on the left) segregating into a distinct group, suggesting they are not from a single origin. FastStructure analysis suggests either K = 6 or K = 7 as the best model, both of which leads to some populations being highly admixed (e.g. BI) while others are fairly homogenous (e.g. SH). (DOCX) [file pgen.1008593.s003.docx]
