# Supplementary material for: Parallel and nonparallel genomic responses contribute to herbicide resistance in Ipomoea purpurea, a common agricultural weed
Source: PLoS Genet. 2020 Feb 3;16(2):e1008593. doi: 10.1371/journal.pgen.1008593 (PMC7018220; doi:10.1371/journal.pgen.1008593)

**S4 Fig. RADseq outliers associated with environmental variables.** Based on BayEnv2 analyses using environmental variables, we identified 50 loci that correlated with minimum temperature of coldest month, only 2 of which overlapped with the resistance outliers; 27 loci correlated with precipitation of the driest month, 0 of which overlapped with the resistance outliers; 36 loci correlated with elevation, 0 of which overlapped with the resistance outliers.


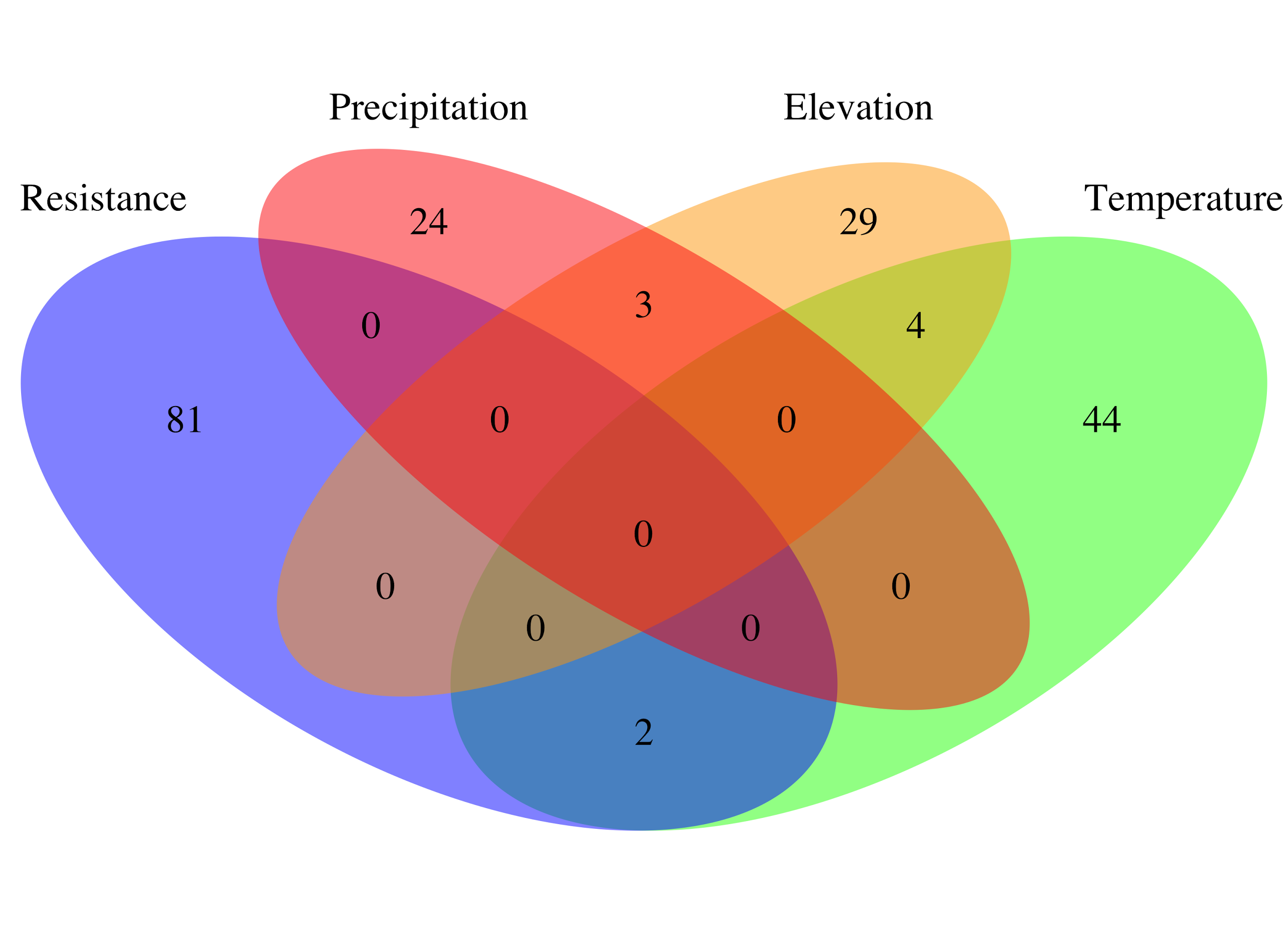

Supplement: S4 Fig — Based on bayenv2 analyses using environmental variables, we identified 50 loci that correlated with minimum temperature of coldest month, only 2 of which overlapped with the resistance outliers; 27 loci correlated with precipitation of the driest month, 0 of which overlapped with the resistance outliers; 36 loci correlated with elevation, 0 of which overlapped with the resistance outliers. (DOCX) [file pgen.1008593.s004.docx]
