# Supplementary material for: Parallel and nonparallel genomic responses contribute to herbicide resistance in Ipomoea purpurea, a common agricultural weed
Source: PLoS Genet. 2020 Feb 3;16(2):e1008593. doi: 10.1371/journal.pgen.1008593 (PMC7018220; doi:10.1371/journal.pgen.1008593)
Supplement: S5 Fig — A screen for outliers when resistance status was randomly assigned to populations (100 randomizations) did not uncover the same pattern of enrichment across chromosomes as the original outlier screen. None of the randomizations produced 5 outlier regions (A). Few randomizations led to a greater number of outliers (B; vertical line denotes the number of outliers found in the original grouping). The regions didn’t show an increased frequency of being identified as outliers in the randomly assigned analysis (C; y-axis shows the number of times the SNP was identified as an outlier in the randomizations). In Fig C, the fifteen chromosomes are presented in different colors on the x-axis (as in Fig 2A) and the greyed areas represent the regions showing outlier enrichment from the original screen. (DOCX) [file pgen.1008593.s005.docx]

**S5 Fig. RADseq outliers when resistance/susceptible status was randomly assigned to populations.** A screen for outliers when resistance status was randomly assigned to populations (100 randomizations) did not uncover the same pattern of enrichment across chromosomes as the original outlier screen. None of the randomizations produced 5 outlier regions (A). Few randomizations led to a greater number of outliers (B; vertical line denotes the number of outliers found in the original grouping). The regions didn’t show an increased frequency of being identified as outliers in the randomly assigned analysis (C; y-axis shows the number of times the SNP was identified as an outlier in the randomizations). In Fig C, the fifteen chromosomes are presented in different colors on the x-axis (as in Fig 2A) and the greyed areas represent the regions showing outlier enrichment from the original screen.

A.


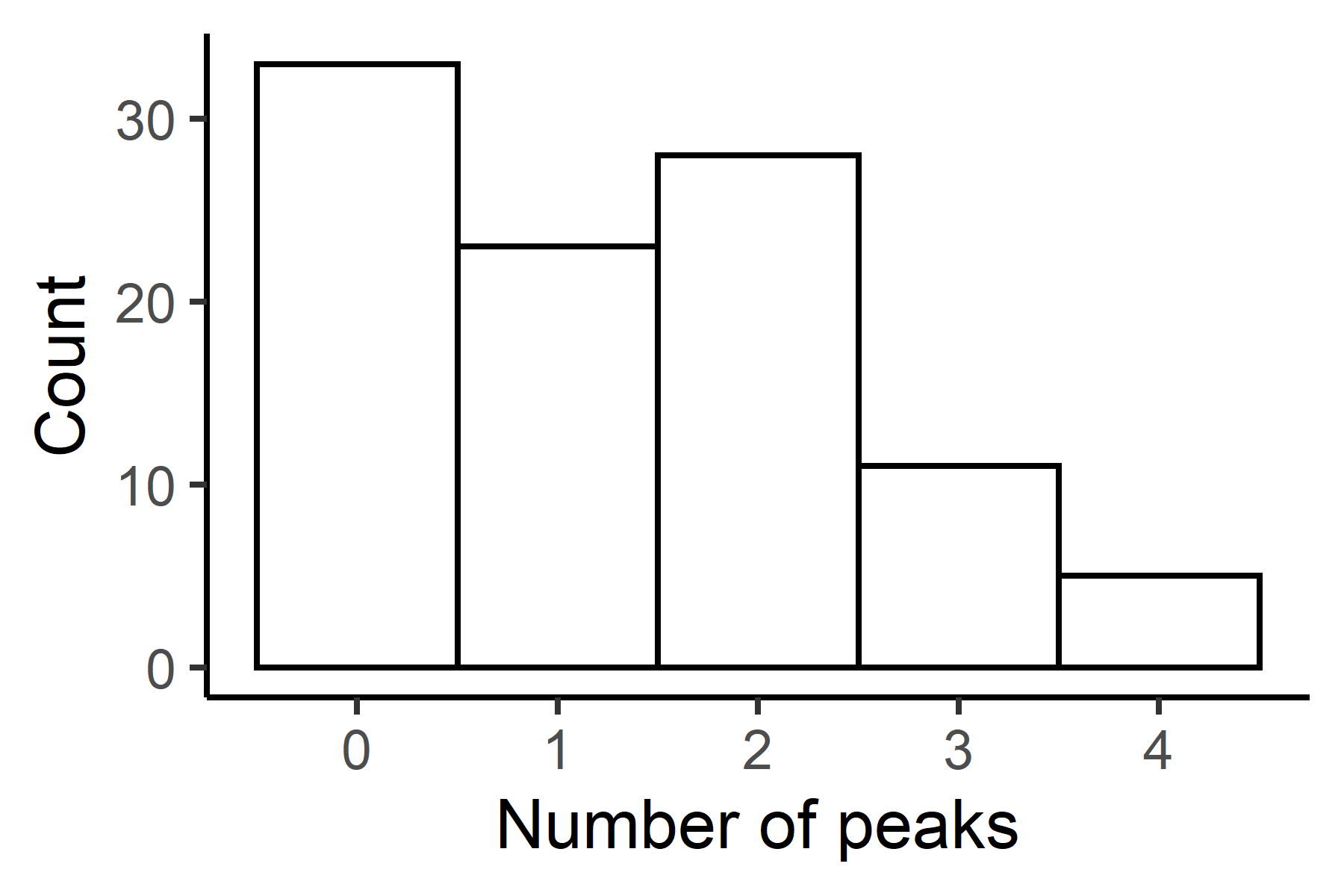


B.


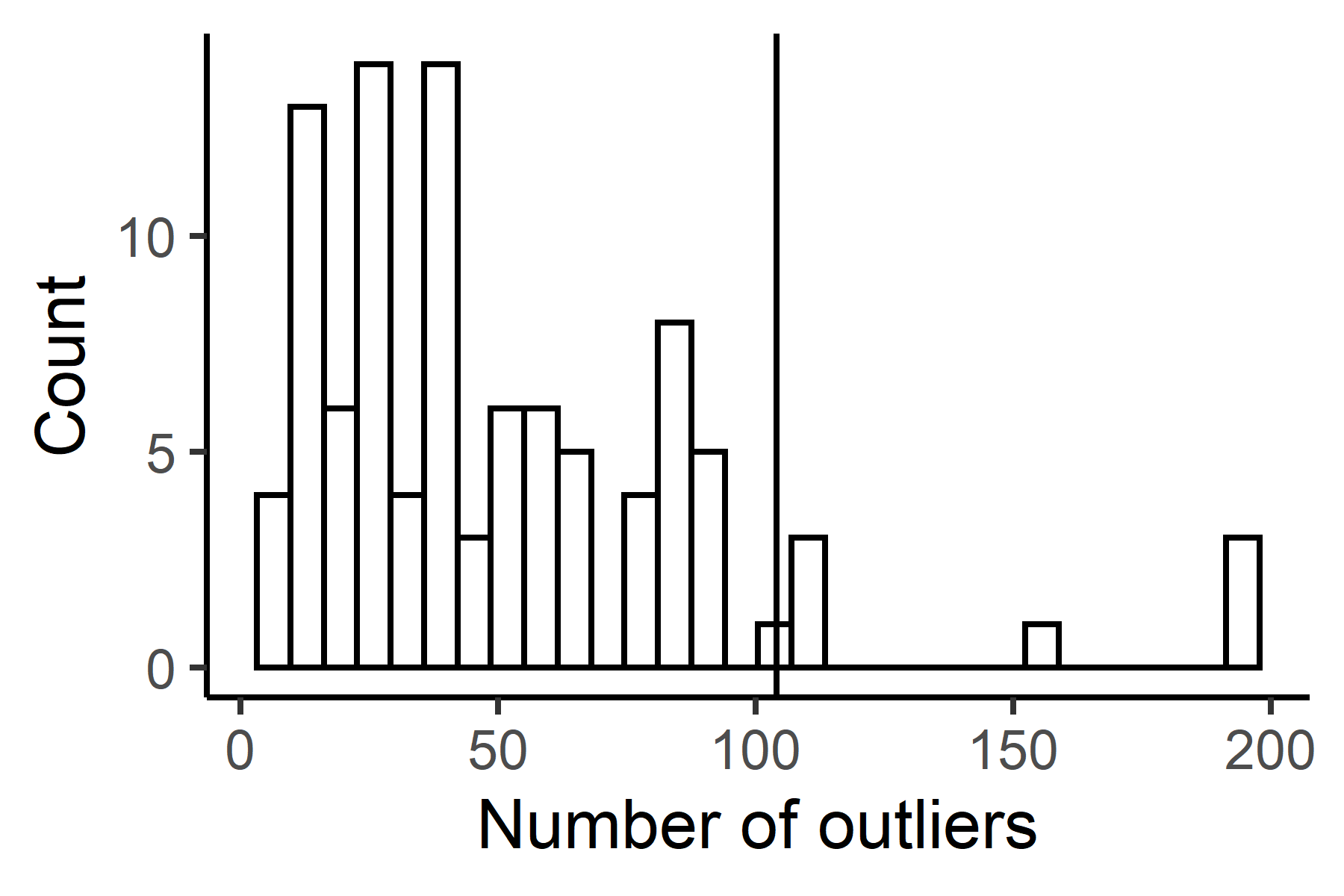


C.

**
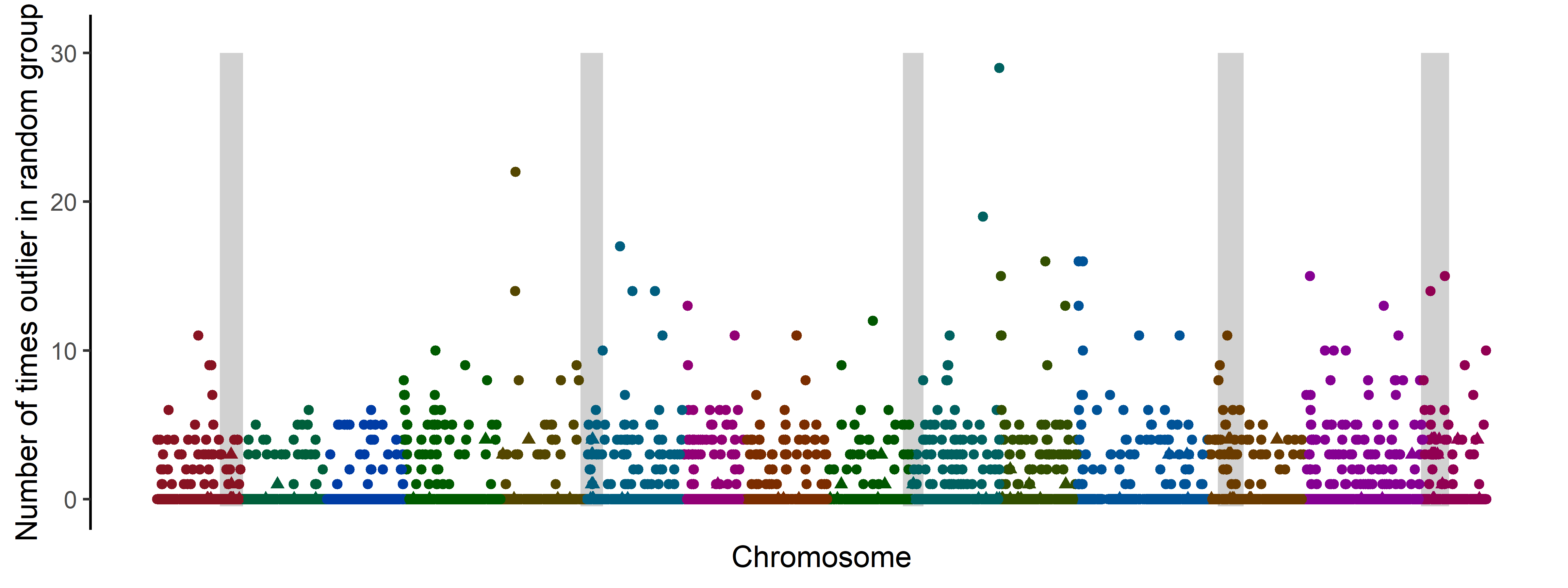
**
