# Supplementary material for: Parallel and nonparallel genomic responses contribute to herbicide resistance in Ipomoea purpurea, a common agricultural weed
Source: PLoS Genet. 2020 Feb 3;16(2):e1008593. doi: 10.1371/journal.pgen.1008593 (PMC7018220; doi:10.1371/journal.pgen.1008593)

**S6 Fig.** **Differences between outliers inside and outside of outlier enriched regions.** (A-D) Distributions of the number of genes within 4 mb of an outlier, either inside (blue) or outside (red) an outlier-enriched region. For each type of gene, the outliers outside of the regions show a left-skewed distribution indicating fewer close detoxification genes for (A) ABC transporters, (B) Glycosyltransferases, (C) Cytochrome P450s and (D) Glutathione S-transferases. (E) Outliers outside of the regions have lower frequencies of the resistant haplotype than those inside the regions, suggesting they are more population specific.


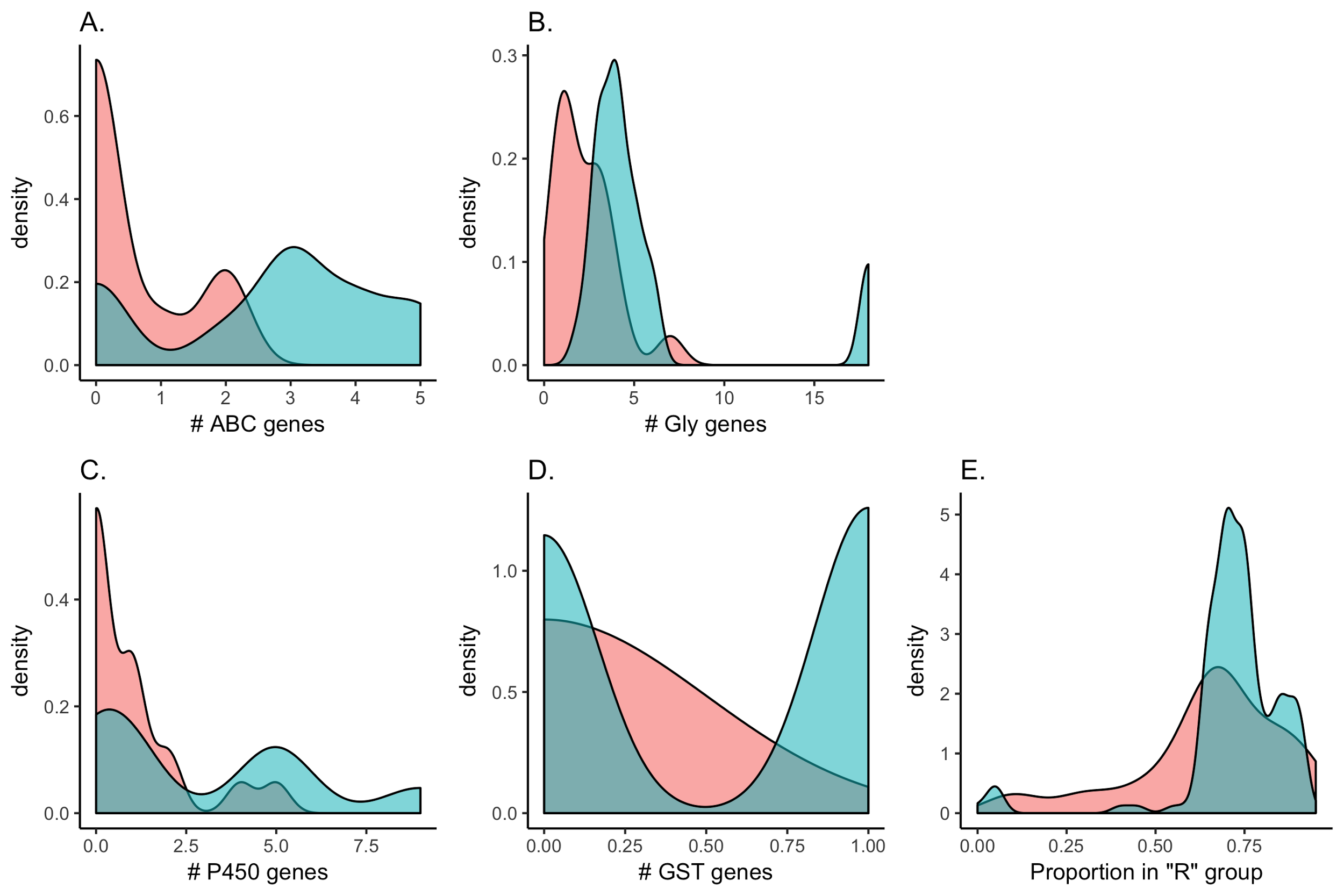

Supplement: S6 Fig — (A-D) Distributions of the number of genes within 4 mb of an outlier, either inside (blue) or outside (red) an outlier-enriched region. For each type of gene, the outliers outside of the regions show a left-skewed distribution indicating fewer close detoxification genes for (A) ABC transporters, (B) Glycosyltransferases, (C) Cytochrome P450s and (D) Glutathione S-transferases. (E) Outliers outside of the regions have lower frequencies of the resistant haplotype than those inside the regions, suggesting they are more population specific. (DOCX) [file pgen.1008593.s006.docx]
