# Supplementary material for: Parallel and nonparallel genomic responses contribute to herbicide resistance in Ipomoea purpurea, a common agricultural weed
Source: PLoS Genet. 2020 Feb 3;16(2):e1008593. doi: 10.1371/journal.pgen.1008593 (PMC7018220; doi:10.1371/journal.pgen.1008593)

**S7. Fig Nucleotide diversity across all SNPs that aligned to the *I. nil* genome.** Data are shown are the ratio of susceptible to resistant individual nucleotide diversity. Grey bars indicate the outlier enriched regions identified on chromosomes 1, 6, 10, 13, and 15. Dashed lines show the 5% most extreme genome-wide values.


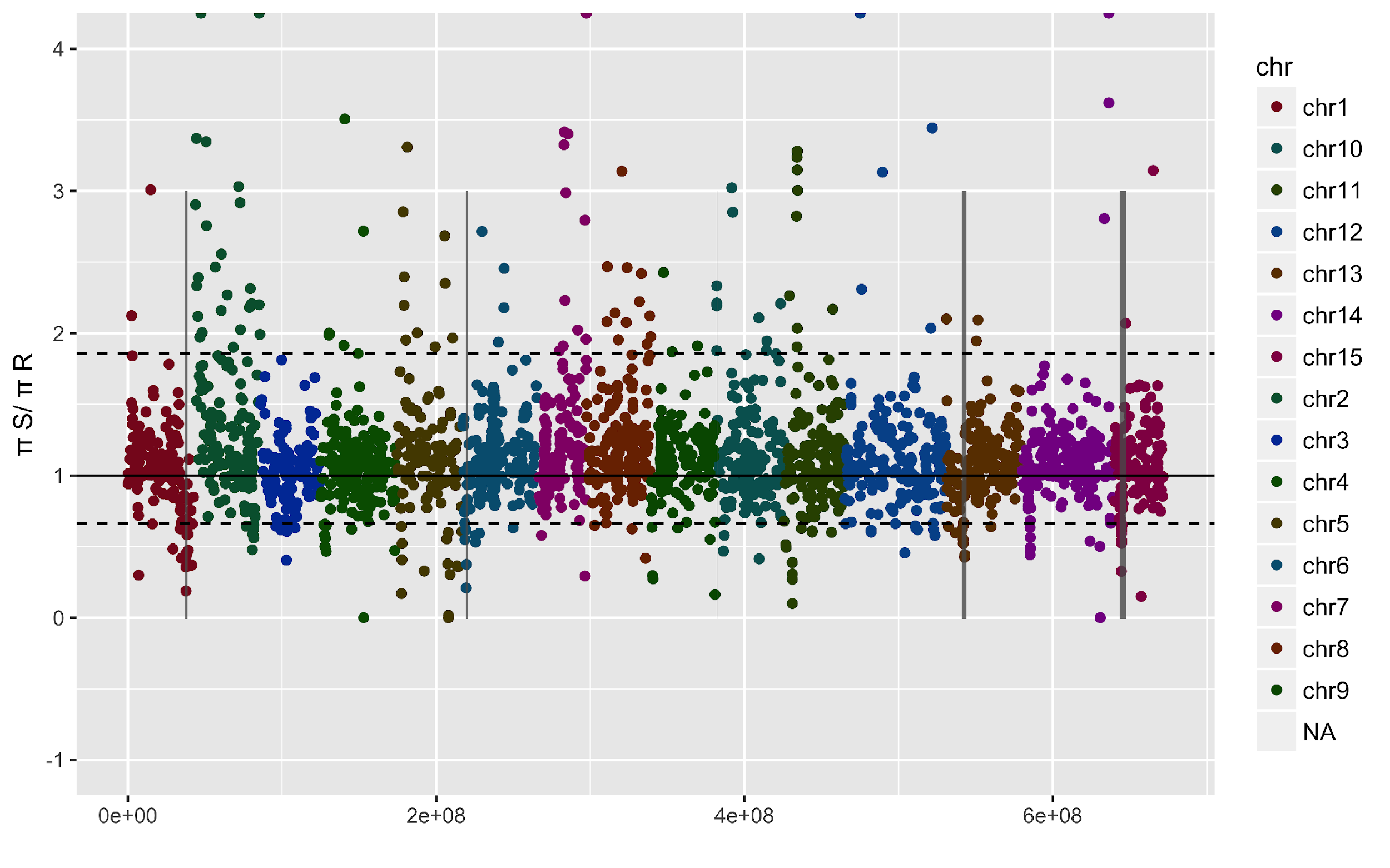

Supplement: S7 Fig — Data are shown are the ratio of susceptible to resistant individual nucleotide diversity. Grey bars indicate the outlier enriched regions identified on chromosomes 1, 6, 10, 13, and 15. Dashed lines show the 5% most extreme genome-wide values. (DOCX) [file pgen.1008593.s007.docx]
