# Supplementary material for: Parallel and nonparallel genomic responses contribute to herbicide resistance in Ipomoea purpurea, a common agricultural weed
Source: PLoS Genet. 2020 Feb 3;16(2):e1008593. doi: 10.1371/journal.pgen.1008593 (PMC7018220; doi:10.1371/journal.pgen.1008593)
Supplement: S8 Fig — (A) Likelihood ratio of the following models relative to a neutral model with no selection: standing variant model (blue), migration (green) or independent mutation (red). (B) Likelihood surface for minimum frequency of the standing variant and the strength of selection holding the age of the standing variant constant; the point indicates the highest likelihood; the color indicates the likelihood (white (high) to yellow to red (low)). (C) Likelihood for the minimum age of standing variant maximizing over the other parameters. (DOCX) [file pgen.1008593.s008.docx]

**S8 Fig. Test of convergence across outlier enriched region for each chromosome .** (A) Likelihood ratio of the following models relative to a neutral model with no selection: standing variant model (blue), migration (green) or independent mutation (red). (B) Likelihood surface for minimum frequency of the standing variant and the strength of selection holding the age of the standing variant constant; the point indicates the highest likelihood; the color indicates the likelihood (white (high) to yellow to red (low)). (C) Likelihood for the minimum age of standing variant maximizing over the other parameters.

**
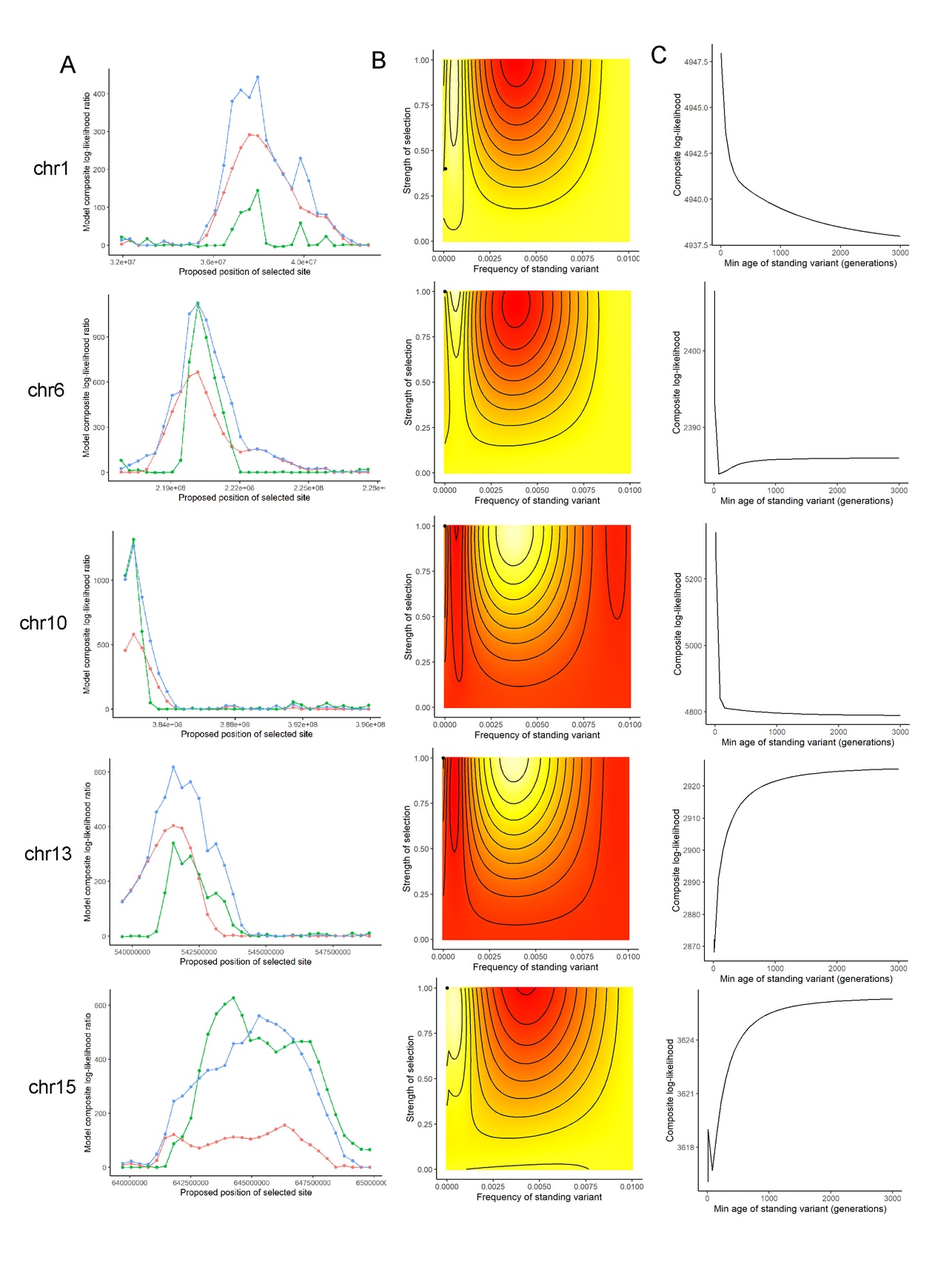
**
