# Supplementary material for: Parallel and nonparallel genomic responses contribute to herbicide resistance in Ipomoea purpurea, a common agricultural weed
Source: PLoS Genet. 2020 Feb 3;16(2):e1008593. doi: 10.1371/journal.pgen.1008593 (PMC7018220; doi:10.1371/journal.pgen.1008593)
Supplement: S1 Table — SNP # = the location of the SNP after alignment with EPSPS from Convulvulus arvensis, Ho = observed heterozygosity (across all samples), He = expected heterozygosity, HWE p-value = p-value for test of Hardy-Weinberg equilibrium from permutation test, Alleles = SNP alleles, Syn = whether a synonymous change (as determined by alignment with C. arvensis sequence), P-value chi-squared R vs S = p-value for test of allele frequency difference between resistant and susceptible populations, P-value cor with resistance = adjusted p-value for correlation with survival. (DOCX) [file pgen.1008593.s009.docx]

**S1 Table.** *EPSPS* SNP data for gene copy A and B. SNP #=the location of the SNP after alignment with *EPSPS* from *Convulvulus arvensis*, Ho=observed heterozygosity (across all samples), He=expected heterozygosity, HWE p-value=p-value for test of Hardy-Weinberg equilibrium from permutation test, Alleles=SNP alleles, Syn=whether a synonymous change (as determined by alignment with *C. arvensis* sequence), P-value chi-squared R vs S=p-value for test of allele frequency difference between resistant and susceptible populations, P-value cor with resistance=adjusted p-value for correlation with survival.

| SNP # | Ho | He | HWE p-value | Alleles | Syn | P-value chi-squared R vs S | P-value cor with resistance |
| --- | --- | --- | --- | --- | --- | --- | --- |
| *EPSPS A* | | | | | | | |
| 102 | 0.19 | 0.47 | 0 | A:G | Y | 0.68 | 0.20 |
| 188 | 0.02 | 0.02 | 1 | A:G | N | 0.89 | 0.45 |
| 234 | 0.2 | 0.47 | 0 | C:G | Y | 0.68 | 0.19 |
| 247 | 0.2 | 0.47 | 0 | C:T | Y | 0.68 | 0.19 |
| 265 | 0.02 | 0.02 | 1 | A:G | N | 0.89 | 0.45 |
| 689 | 0.11 | 0.11 | 1 | A:T | N | 0.77 | 0.12 |
| 690 | 0.11 | 0.11 | 1 | C:A | N | 0.77 | 0.12 |
| 741 | 0.16 | 0.48 | 0 | G:A | Y | 0.64 | 0.19 |
| 831 | 0.18 | 0.47 | 0 | C:T | Y | 0.66 | 0.19 |
| 936 | 0.2 | 0.48 | 0 | T:C | Y | 0.66 | 0.20 |
| 1194 | 0.21 | 0.48 | 0.001 | T:C | Y | 0.69 | 0.21 |
| 1425 | 0.18 | 0.49 | 0 | A:G | Y | 0.67 | 0.19 |
| 1500 | 0.11 | 0.5 | 0 | T:G | Y | 0.57 | 0.19 |
| 1503 | 0.14 | 0.5 | 0 | T:C | Y | 0.62 | 0.19 |
| *EPSPS B* | | | | | | | |
| 214 | 0.18 | 0.42 | 0.001 | G:A | N | 0.85 | 0.59 |
| 246 | 0.18 | 0.4 | 0 | T:G | Y | 0.84 | 0.59 |
| 372 | 0.18 | 0.42 | 0.001 | C:T | Y | 0.80 | 0.59 |
| 496 | 0.18 | 0.42 | 0 | C:A | N | 0.85 | 0.59 |
| 497 | 0.27 | 0.63 | 0 | T:C:G | N | 0.89 | 0.17 |
| 606 | 0.18 | 0.4 | 0.001 | T:G | Y | 0.86 | 0.59 |
| 666 | 0.18 | 0.4 | 0 | T:C | Y | 0.84 | 0.59 |
| 729 | 0.23 | 0.5 | 0.001 | T:A | Y | 0.89 | 0.45 |
| 792 | 0.16 | 0.31 | 0.002 | C:T | Y | 0.69 | 0.17 |
| 921 | 0.16 | 0.34 | 0.003 | C:T | Y | 0.67 | 0.45 |
| 963 | 0.16 | 0.34 | 0.005 | C:T | Y | 0.67 | 0.45 |
| 1029 | 0.3 | 0.49 | 0.01 | A:G | Y | 1.00 | 0.45 |
| 1034 | 0.3 | 0.49 | 0.007 | G:C | N | 1.00 | 0.45 |
| 1044 | 0.3 | 0.49 | 0.012 | T:C | Y | 1.00 | 0.45 |
| 1053 | 0.18 | 0.35 | 0.002 | C:T | Y | 0.67 | 0.17 |
| 1114 | 0.3 | 0.49 | 0.012 | C:G | N | 0.98 | 0.45 |
| 1278 | 0.02 | 0.49 | 0 | T:A | N | 0.97 | 0.48 |
| 1359 | 0.16 | 0.34 | 0.002 | C:G | Y | 0.67 | 0.45 |
| 1392 | 0.3 | 0.49 | 0.011 | A:G | Y | 1.00 | 0.45 |
| 1401 | 0.3 | 0.49 | 0.022 | G:A | Y | 1.00 | 0.45 |
| 1413 | 0.16 | 0.34 | 0.001 | A:G | Y | 0.67 | 0.45 |
| 1503 | 0.02 | 0.02 | 1 | C:T | Y | 0.89 | 0.66 |
