# Supplementary material for: Parallel and nonparallel genomic responses contribute to herbicide resistance in Ipomoea purpurea, a common agricultural weed
Source: PLoS Genet. 2020 Feb 3;16(2):e1008593. doi: 10.1371/journal.pgen.1008593 (PMC7018220; doi:10.1371/journal.pgen.1008593)
Supplement: S2 Table — Population = population abbreviation. Ave N/locus = average number of individuals with high quality allele data per locus. % loci missing = average percent of the population with missing data per locus. Ho = observed heterozygosity. He = expected heterozygosity. FIS = Wright’s inbreeding coefficient. (DOCX) [file pgen.1008593.s010.docx]

**S2 Table.** Population genetics parameters for the RADseq SNPs. Population = population abbreviation. Ave N/locus = average number of individuals with high quality allele data per locus. % loci missing = average percent of the population with missing data per locus. Ho = observed heterozygosity. He = expected heterozygosity. FIS = Wright’s inbreeding coefficient.

| Population | Ave N/locus | % loci missing | Ho | He | FIS |
| --- | --- | --- | --- | --- | --- |
| RB | 9.518758 | 0.048124 | 0.239929 | 0.260012 | 0.101951 |
| HA | 9.585627 | 0.041437 | 0.276958 | 0.325516 | 0.135513 |
| BI | 9.223264 | 0.077674 | 0.282875 | 0.335554 | 0.151809 |
| DW | 9.539342 | 0.046066 | 0.242219 | 0.267021 | 0.112749 |
| FL | 9.610597 | 0.03894 | 0.278797 | 0.309048 | 0.107022 |
| SH | 9.648599 | 0.03514 | 0.26598 | 0.247444 | -0.03845 |
| SPC | 9.632156 | 0.036784 | 0.207493 | 0.222291 | 0.119612 |
| WG | 9.488307 | 0.051169 | 0.209562 | 0.251296 | 0.209932 |
