# Supplementary material for: Parallel and nonparallel genomic responses contribute to herbicide resistance in Ipomoea purpurea, a common agricultural weed
Source: PLoS Genet. 2020 Feb 3;16(2):e1008593. doi: 10.1371/journal.pgen.1008593 (PMC7018220; doi:10.1371/journal.pgen.1008593)
Supplement: S3 Table — (DOCX) [file pgen.1008593.s011.docx]

**S3 Table.** Assembly statistics for the Illumina genome assembly (using ABYSS-PE), the PacBio + Illumina genome assembly (using DBLOG2), the resequencing assembly (using Megahit) and the resequencing assembly contigs containing SNPs.

|  | Illumina | PacBio+Illumina | Denovo contigs | Denovo contigs with SNPs |
| --- | --- | --- | --- | --- |
| Number of contigs | 1933851 | 17897 | 67266 | 26988 |
| Smallest contig | 64 | 231 | 200 | 200 |
| Largest Contig | 94914 | 162047 | 16167 | 16167 |
| Number of bases | 631125096 | 194706849 | 29298709 | 13126985 |
| Mean contig length | 237.49186 | 10879.30094 | 435.56 | 486.40 |
| n_under_200 | 1679726 | 0 | 0 | 0 |
| Number of contigs over 1k | 107943 | 17846 | 1456 | 832 |
| Number of contigs over 10k | 5686 | 6597 | 3 | 2 |
| n90 | 809 | 5106 | 268 | 301 |
| n70 | 2774 | 9988 | 363 | 415 |
| n50 | 6790 | 15425 | 458 | 530 |
| n30 | 23809 | 25478 | 592 | 668 |
| n10 | 94914 | 49505 | 908 | 975 |
| gc% | 0.37927 | 0.38219 | 0.44 | 0.45 |
| Number of bases that are N | 361257 | 439633 | 0 | 0 |
| Proportion of bases that are N | 0.00057 | 0.00226 | 0 | 0 |
