# Supplementary material for: Parallel and nonparallel genomic responses contribute to herbicide resistance in Ipomoea purpurea, a common agricultural weed
Source: PLoS Genet. 2020 Feb 3;16(2):e1008593. doi: 10.1371/journal.pgen.1008593 (PMC7018220; doi:10.1371/journal.pgen.1008593)
Supplement: S5 Table — (DOCX) [file pgen.1008593.s013.docx]

**S5 Table.** Summary of SNPs used in the analysis of linkage disequilibrium in the regions enriched for outliers, per chromosome, as identified by bayenv2 or Bayescan. Only SNPs that could be mapped to the genome of the close relative, *I. nil*, were used in analyses. r^2^ values were determined using all individuals regardless of population or resistance level.

| Chromosome | SNP Number | r^2^ mean | SD | r^2^ 75^%^ | Size of region with outliers | Size of region with high LD,  r^2^ > 0.25 |
| --- | --- | --- | --- | --- | --- | --- |
| 1 | 76 | 0.313 | 0.302 | 0.475 | 1.56MB | ~1MB |
| 6 | 54 | 0.251 | 0.283 | 0.334 | 1.37MB | 0.84MB |
| 10 | 20 | 0.880 | 0.169 | 0.950 | 276KB | 0.94MB |
| 13 | 91 | 0.208 | 0.334 | 0.190 | 2.9MB | 1.55MB |
| 15 | 195 | 0.205 | 0.314 | 0.288 | >4MB | ~3MB |
