# Supplementary material for: Parallel and nonparallel genomic responses contribute to herbicide resistance in Ipomoea purpurea, a common agricultural weed
Source: PLoS Genet. 2020 Feb 3;16(2):e1008593. doi: 10.1371/journal.pgen.1008593 (PMC7018220; doi:10.1371/journal.pgen.1008593)
Supplement: S6 Table — BI, DW, SPC, and WG are the high resistance populations. (DOCX) [file pgen.1008593.s014.docx]

**S6 Table.** Neutral F matrix from scaffolds on chromosomes 3, 7, and 14 (61 scaffolds total). BI, DW, SPC, and WG are the high resistance populations.

|  | BI | DW | SPC | WG | RB | HA | FL | SH |
| --- | --- | --- | --- | --- | --- | --- | --- | --- |
| BI | 0.223 | 0.180 | 0.181 | 0.068 | 0.209 | 0.159 | 0.080 | 0.140 |
| DW | 0.180 | 0.404 | 0.177 | 0.075 | 0.260 | 0.271 | 0.097 | 0.158 |
| SPC | 0.181 | 0.177 | 0.428 | 0.083 | 0.202 | 0.188 | 0.098 | 0.137 |
| WG | 0.068 | 0.075 | 0.083 | 0.484 | 0.034 | 0.095 | 0.000 | 0.001 |
| RB | 0.209 | 0.260 | 0.202 | 0.034 | 0.420 | 0.184 | 0.027 | 0.178 |
| HA | 0.159 | 0.271 | 0.188 | 0.095 | 0.184 | 0.295 | 0.098 | 0.062 |
| FL | 0.080 | 0.097 | 0.098 | 0.000 | 0.027 | 0.098 | 0.131 | 0.010 |
| SH | 0.140 | 0.158 | 0.137 | 0.001 | 0.178 | 0.062 | 0.010 | 0.322 |
