# Supplementary material for: Parallel and nonparallel genomic responses contribute to herbicide resistance in Ipomoea purpurea, a common agricultural weed
Source: PLoS Genet. 2020 Feb 3;16(2):e1008593. doi: 10.1371/journal.pgen.1008593 (PMC7018220; doi:10.1371/journal.pgen.1008593)
Supplement: S7 Table — (DOCX) [file pgen.1008593.s015.docx]

**S7 Table**. Parameter spaces for composite-likelihood calculations for the standing variation (s, t, g) and migration (s, m, source population) model simulations.

| Position of selected site | 37189, 37198, 37224, 37246, 37258, 37267, 37271, 37273, 37282, 37283, 37285, 37288, 37303, 37305, 37342, 37355, 37357, 37360, 37362, 37366, 37376, 37408, 140310, 140466, 140544, 140552, 140565, 140571, 140605, 140627 |
| --- | --- |
| s | 0.001, 0.002, 0.003, 0.004, 0.005, 0.006, 0.007, 0.008, 0.009, 0.01, 0.011, 0.014, 0.016, 0.019, 0.021, 0.024, 0.026, 0.029, 0.032, 0.034, 0.037 0.039, 0.042, 0.045, 0.047, 0.05, 0.052, 0.055, 0.057, 0.06, 0.08, 0.1, 0.15, 0.2, 0.25, 0.3, 0.35, 0.4, 0.45, 0.5, 0.55, 0.6, 0.65, 0.7, 0.75, 0.8, 0.85, 0.9, 0.95, 1 |
| t | 5, 10, 81, 151, 222, 293, 364, 434, 505, 576, 646, 717, 788, 859, 929, 1000, 1500, 1607, 1714, 1821, 1929, 2036, 2143, 2250, 2357, 2464, 2571, 2679, 2786, 2893, 3000 |
| g | 10^−10^, 10^−9^ , 10^−8^ , 10^−7^ , 10^−6^ , 10^−5^ , 10^−4^ , 10^−3^ , 10^−2^ |
| m | 10^−5^ , 10^−4^ , 5^−4^ , 0.001, 0.005, 0.01, 0.1, 0.2 0.3, 0.4, 0.5, 0.6, 0.7, 0.8, 0.9, 1 |
| source population | SPC and WG |
