# Supplementary material for: Parallel and nonparallel genomic responses contribute to herbicide resistance in Ipomoea purpurea, a common agricultural weed
Source: PLoS Genet. 2020 Feb 3;16(2):e1008593. doi: 10.1371/journal.pgen.1008593 (PMC7018220; doi:10.1371/journal.pgen.1008593)
Supplement: S8 Table — Maximum log-likelihood values for the independent mutation, migration, and standing variation model, along with the position (bp) at which the maximum log-likelihood was achieved. The maximum selection coefficient (s), frequency of the variant (g), and minimum standing variation time prior to the onset of selection (t) were calculated for the standing variation model. (DOCX) [file pgen.1008593.s016.docx]

**S8 Table**. **Summary of model parameters presented in S8 Fig.** Maximum log-likelihood values for the independent mutation, migration, and standing variation model, along with the position (bp) at which the maximum log-likelihood was achieved. The maximum selection coefficient (s), frequency of the variant (g), and minimum standing variation time prior to the onset of selection (t) were calculated for the standing variation model.

| Chromosome | Maximum log likelihood | | | Position with max log likelihood | Max selection coefficient, s | Max frequency, g | Min standing variation time, t |
| --- | --- | --- | --- | --- | --- | --- | --- |
|  | Independent mutations | Migration | Standing ancestral variation |  |  |  |  |
| Chr1 | 291.62 | 144.55 | 443.66 | 37960691 | 0.4 | 1.00E-04 | 5 |
| Chr6 | 665.62 | 1120.08 | 1125.64 | 220167244 | 1 | 1.00E-06 | 5 |
| Chr10 | 581.22 | 1316.62 | 1269.70 | 381993922 | 1 | 1.00E-06 | 5 |
| Chr13 | 404.01 | 339.77 | 617.73 | 541532763 | 1 | 1.00E-06 | 3000 |
| Chr15 | 156.70 | 627.57 | 560.82 | 644244852 | 1 | 1.00E-05 | 3000 |
