# Supplementary material for: Parallel and nonparallel genomic responses contribute to herbicide resistance in Ipomoea purpurea, a common agricultural weed
Source: PLoS Genet. 2020 Feb 3;16(2):e1008593. doi: 10.1371/journal.pgen.1008593 (PMC7018220; doi:10.1371/journal.pgen.1008593)
Supplement: S1 Dataset — (DOCX) [file pgen.1008593.s017.docx]

**S1 Dataset:**

Tables include annotations of outlier RADseq loci, annotations of probe sequences used for target capture probes, annotation of outlier contigs from resequencing, a list of *I. nil* genes within the 5 outlier enriched regions, and EPSPS primer sequences.

<https://docs.google.com/spreadsheets/d/1I59RoHSTc4ktXMOuZQuN5KNxMQ0Lprozqma8Cf3gBmA/edit?usp=sharing>

The folder includes input and output files for bayenv2 and BayeScan analyses (list below).

<https://drive.google.com/open?id=1fLVjN8HnFm6Rffbo_rAV-bJieBapqABx>

Target capture bayenv2:

TC_BE_output.bf = output file

TC_BE_input.be.txt = input file

TC_BE_matrix.txt = matrix

RadSeq bayenv2:

Rad_BE_matrix.txt

Rad_BE_input.txt

Rad_BE_output.txt

Target capture BayeScan:

TC_BS_output.txt

TC_BS_input.bs

RadSeq BayeScan:

Rad_BS_input.bs

Rad_BS_output.txt
